# Characteristic of Motor Control in Three-Dimensional Circular Tracking Movements during Monocular Vision

**DOI:** 10.1155/2019/3867138

**Published:** 2019-11-03

**Authors:** Woong Choi, Liang Li, Jongho Lee

**Affiliations:** ^1^Department of Information and Computer Engineering, National Institute of Technology Gunma College, Maebashi 371-8530, Japan; ^2^College of Information Science and Engineering, Ristumeikan University, Kusatsu 525-8577, Japan; ^3^Department of Clinical Engineering, Komatsu University, Komatsu 923-8511, Japan

## Abstract

Analysis of visually guided tracking movements is an important component of understanding human visuomotor control system. The aim of our study was to investigate the effects of different target speeds and different circular tracking planes, which provide different visual feedback of depth information, on temporal and spatial tracking accuracy. In this study, we analyze motor control characteristic of circular tracking movements during monocular vision in three-dimensional space using a virtual reality system. Three parameters in polar coordinates were analyzed: Δ*R*, the difference in the distance from the fixed pole; Δ*θ*, the difference in the position angle; and Δ*ω*, the difference in the angular velocity. We compare the accuracy of visually guided circular tracking movements during monocular vision in two conditions: (1) movement in the frontal plane relative to the subject that requires less depth information and (2) movement in the sagittal plane relative to the subject that requires more depth information. We also examine differences in motor control at four different target speeds. The results show that depth information affects both spatial and temporal accuracy of circular tracking movement, whereas target speed only affects temporal accuracy of circular tracking movement. This suggests that different strategies of feedforward and feedback controls are performed in the tracking of movements.

## 1. Introduction

Visually guided tracking of movement is an important mechanism for learning skills using the visuomotor system such as watching and imitating the movement of others in sports and dancing [1–5]. Unlike reaching movements, tracking movement requires feedback control based on the perception of velocity and depth using visuomotor targets with spatiotemporal fluctuation. Research has focused on analyzing control characteristic such as feedforward and feedback mechanisms mediated through visual information from target movement.

Research into visually guided tracking of movement has focused on the task of tracking a visually guided target with a trajectory, in a one-dimensional straight line or a two-dimensional plane, through various joint movements in a three-dimensional (3D) space [6–15]. For example, Miall et al. [6–8] examined the task of tracking a visually guided target with a one-dimensional sinusoidal trajectory using the multijoint motion of an arm in a 3D space using both monkeys and humans. They found that control parameters differ depending on the periodicity of the target's orbit in the one-dimensional tracking movement. Also, Beppu et al. [9, 10] performed a tracking task using an elbow joint motion with one degree of freedom in patients with cerebellar disease and normal controls. The targets were visually guided with a one-dimensional ramp trajectory. They discovered parameters that can quantitatively evaluate the severity of cerebellar disease in patients.

In such tracking movements, control elements and evaluation parameters to be tested differ depending on the dimensional range of the target trajectory (i.e., the trajectory on a one-dimensional straight line vs. the trajectory on a two-dimensional plane). It is, therefore, necessary to establish evaluation parameters based on the dimension of the target orbit [16].

Circular tracking movements have similar periodic tracking movements to that of one-dimensional sinusoidal tracking movements. However, unlike one-dimensional tracking movement, constant velocity and continuous movement can be examined in a two-dimensional plane [12–15, 17–19]. These groups tracked targets that had visually guided trajectories on a two-dimensional plane by using a tablet with stylus, a two-dimensional tracer (i.e., computer mouse), and two-degree-of-manipulandum arm and wrist movements. Previously, the field has relied upon measuring and analyzing tracking movement with arms and wrists that can be realized in 3D space by using a visual display in a two-dimensional plane and using a two-dimensional measuring device.

There are many studies that have compared the characteristic of visuomotor control during binocular and monocular vision using the task of reaching and grasping [20–27]. Visuomotor control during binocular vision has been reported as faster than monocular, with less error and the advantage of setting an initial position [20, 22, 24, 26].

In a task where the target can be seen, feedback control is performed during binocular vision when the subject is tracking circular. However, it has been reported that feedforward control is performed during a task where the target is not visible [12]. During monocular vision, humans cannot recognize depth information as accurately as during binocular vision. However, the relative size, occlusion, perspective, motion parallax, and so on can be used as cues to recognize depth information during monocular vision [28, 29].

In the field of computer vision, depth estimation based on stereo images or motion is a well-studied area [30]. However, depth estimation from a single monocular image is a challenging task and has been paid more attention [31–33]. The computer estimates depth information based on either predefined image features or training data. In deep learning-based methods, the machine learns the relationship between image features and depth information from ground truth images and estimates the depth of an input monocular image based on the trained network [32, 33]. Monocular depth prediction can be applied to practical problems such as 3D modeling, robotics, and automatic driving. Quantitative investigation of human visuomotor control in 3D space during monocular vision may provide greater insight into these areas.

Recently, we developed an experimental 3D system for visuomotor control in a virtual reality (VR) environment [34]. We adopted a circular tracking task to 3D VR space and compared the visuomotor control of 3D circular tracking movements between monocular and binocular vision. We found that circular tracking with binocular vision is more accurate than that with monocular vision, and we observed differences in perception of depth between the two forms of vision in the 3D VR environment. Depth estimation is considered an important control metric for motor control as well as depth perception in 3D space.

Visuomotor control in the frontal *ROT0* and sagittal *ROT90* planes with respect to the velocity of the target by monocular vision has not been studied in the circular tracking movement of a 3D VR environment. Therefore, the following are still unanswered questions:

1. What is the relationship between target speed and depth in 3D target-tracking movements during monocular vision?
2. What is the effect of depth on kinematic parameters, such as position and velocity, during monocular vision?

In this study, we analyzed the motor control characteristics of circular tracking movements during monocular vision in a 3D space using three parameters in polar coordinates: the difference in the distance from the fixed pole (Δ*R*), the difference in the position angle (Δ*θ*), and the difference in the angular velocity (Δ*ω*). We investigated the differences in these parameters between circular tracking movements in the frontal and sagittal planes relative to the subject. We also examined variations in motor control at four different target speeds based on these parameters in 3D target-tracking movements during monocular vision.

## 2. Materials and Methods

### 2.1. Subjects and Experimental Setup

The subjects were 15 males with a mean age of 20.1 ± 0.64 years. All had a normal or corrected-to-normal vision. No subjects had previously participated in similar studies. All subjects gave written informed consent before their participation. All experiments were conducted in accordance with relevant guidelines and regulations. The protocol was approved by the Ethics Committee of the National Institute of Technology, Gunma College.

**Figure 1:**
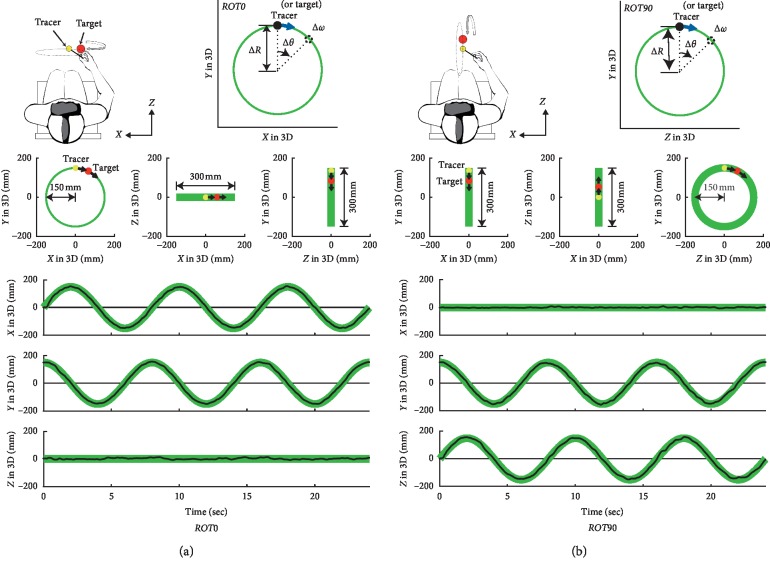
Experimental procedure. (a) Schematic of the circular tracking experiment for the body's frontal plane (*ROT0*). (b) Schematic of the circular tracking experiment for the body's sagittal plane (*ROT90*). Green lines indicate the target's path in the 3D VR space. The three graphs in the middle show the target's path as seen from the front (left), above (center), and the side (right) from the subject's viewpoint. The target's path was not displayed to the subjects during the experiment. The three lower graphs show a typical trial of the target's path (green line) and the tracer's path (black line) for each axis versus time. Insets in the upper-right of (a) and (b) show that how three outcome measures (∆*R*, ∆*θ*, and ∆*ω*) were derived from the path data of the target (or the tracer) for each plane.

The subjects were asked to perform a visually guided tracking task in a 3D VR environment during monocular vision, which involved tracking a target with a tracer (Figure 1). The target was a virtual red ball with a radius of 1.5 cm. The subjects hold the handle of the controller during the experiment. The handle of the controller is displayed as a virtual stick (20 cm long). The direction of the controller was synchronized with that of the virtual stick. In this research, the circular tracking was performed without displaying the 3D hand and 3D arm in VR. The tracer, which was a virtual yellow ball with a radius of 1 cm, was placed at the tip of the stick. The tracer position was synchronized with the subject's hand movements. During the experiment, the target moved continuously along an invisible circular orbit with a radius of 15 cm. The rotation axis was set to two orientations according to the experimental requirements.

All subjects had a normal or corrected-to-normal vision with binocular vision greater than 0.7. The visual acuity of subjects was referred to the results of their health examinations. In this study, we performed an additional examination to evaluate the stereo acuity of the subjects in the VR space. The display position of the target was calibrated for each subject before the examination of stereo acuity. Firstly, whether stereoscopic vision can be properly perceived was confirmed orally. For the participants who could not correctly perceive the target, interocular distance for each subject was accordingly adjusted. Next, stereo acuity was evaluated by a task that puts the tracer into the center of the 3D target. The subjects who succeeded over four times by executing the task five times participated in this research.

### 2.2. Movement Task

In this study, we performed an experiment to quantitatively evaluate 3D visuomotor control, using circular tracking movements for the frontal and the sagittal planes relative to the subject in VR space (Figure 1). The subject's nondominant eye was covered with an eye patch to produce monocular vision. The subjects were seated in a chair built for the experiment and wore a head-mounted display. We confirmed orally before the experiment that each subject could correctly perceive depth information during monocular vision by the size change and occlusion between the target and tracer balls in the 3D space. Subjects were asked to hold the physical controller in their dominant hand. We ran a calibration to locate the target's initial position. The target rotated at 0.125, 0.25, 0.5, or 0.75 Hz along the orbit after a countdown of 3 s with sound effects. The subjects were asked to move the tracer to the target's position during the countdown and then perform a circular tracking movement. As shown in Figure 1, the target stopped after three loops in one trial. One trial finished with a sound effect after the target stopped for one second. Four trials were performed with the target rotating in the frontal plane (*ROT0* in Figure 1(a)) and four with it rotating in the sagittal plane (*ROT90* in Figure 1(b)). Therefore, for each subject, 32 trials were carried out in total (4 trials × 4 speeds × 2 planes). The first trial for each setting was discarded from the analysis to account for adjustment of the subject to the protocol.

### 2.3. Data Analysis

During the movement task, we recorded the positions of the tracer and the target in Cartesian coordinates of 3D VR space at a 90 Hz sampling rate. For the data analysis, we transformed the Cartesian (*X*, *Y*, and *Z*) data to radial displacement, angular displacement, and angular velocity on polar coordinates, and named “*R*,” “*θ*,” and “*ω*,” respectively (see upper-right insets in Figures 1(a) and 1(b)).

∆*R* is defined as the absolute value of the radial position difference between the target and the tracer from the origin as follows:

(1) $$\begin{array}{c} \Delta R=\left|R_{\text{tracer}}-R_{\text{target}}\right|. \end{array}$$

∆*θ* is also defined as the absolute value of the angular displacement difference between the target and the tracer as follows:

(2) $$\begin{array}{c} \Delta \theta =\left|\theta_{\text{tracer}}-\theta_{\text{target}}\right|. \end{array}$$

Δ*ω* denotes the absolute value of the angular velocity difference between the target and the tracer as follows:

(3) $$\begin{array}{c} \Delta \omega =\left|\omega_{\text{tracer}}-\omega_{\text{target}}\right|. \end{array}$$

In this study, we investigated the differences in the parameters of Δ*R*, Δ*θ*, and Δ*ω* between circular tracking movements on the frontal and sagittal planes in monocular vision condition.

For analyzing the differences in circular tracking movements based on Δ*R*, Δ*θ*, and Δ*ω*, we carried out a two-way repeated-measures analysis of variance (ANOVA), with the plane factor (with two levels: *ROT0*, frontal plane; *ROT90*, sagittal plane) and the speed factor (with four levels: *V*1, 0.125 Hz (*n* = 15); *V*2, 0.25 Hz (*n* = 15); *V*3, 0.5 Hz (*n* = 15); and *V*4, 0.75 Hz (*n* = 15)). The main effects and interaction of plane and speed factors in the parameters of Δ*R*, Δ*θ*, and Δ*ω* were assessed by the Repeated Measures function in SPSS Statistics, IBM. We performed Mauchly's sphericity test (SPSS Statistics, IBM) to validate the result of ANOVA. If sphericity was assumed (*p* > 0.05), the values corrected with sphericity assumed in tests of within-subjects effects were used. If sphericity was not assumed (*p* < 0.05), the values corrected with Greenhouse–Geisser in tests of within-subjects effects were used.

The post hoc test was conducted by the pairwise comparisons of Bonferroni correction. Except where noted, we describe data using the mean (*M*), standard error (SE), and standard deviation (SD). We considered comparisons yielding *p* < 0.05 to be statistically significant and comparisons yielding *p* < 0.01 to be highly statistically significant. These outlined methods and statistical analyses were used to produce the data in Tables 1-3.

Also, Pearson's correlation coefficient *r* was used to indicate the effect size of *t*-test. The *r* values of the effect size in Items B, C, and D of Tables 1–3 were calculated by the equation of $\;r\;=\;\sqrt{t^{2}/\left(t^{2}+\text{df}\right)}$, where *t* is the *t* value and df is degrees of freedom [35].

## 3. Results

### 3.1. Differences in Performance Based on ΔR

**Figure 2:**
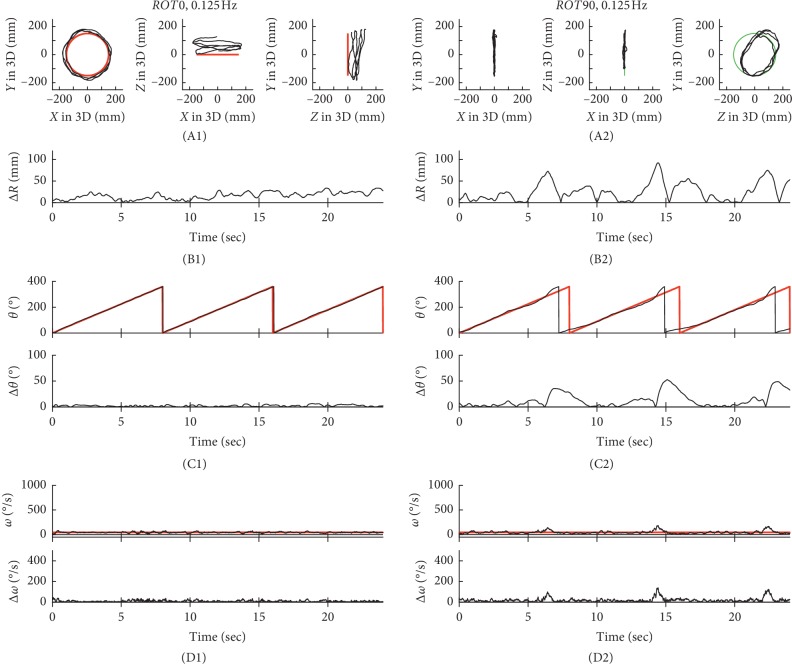
Typical examples of tracking movements at 0.125 Hz. The trajectories of circular tracking movements for 0.125 Hz in the frontal plane (*ROT0*, A1) and the sagittal plane (*ROT90*, A2). Absolute values of Δ*R* for *ROT0* (B1) and *ROT90* (B2). *θ* and absolute values of Δ*θ* for *ROT0* (C1) and *ROT90* (C2). Absolute values of Δ*ω* for *ROT0* (D1) and *ROT90* (D2).

**Figure 3:**
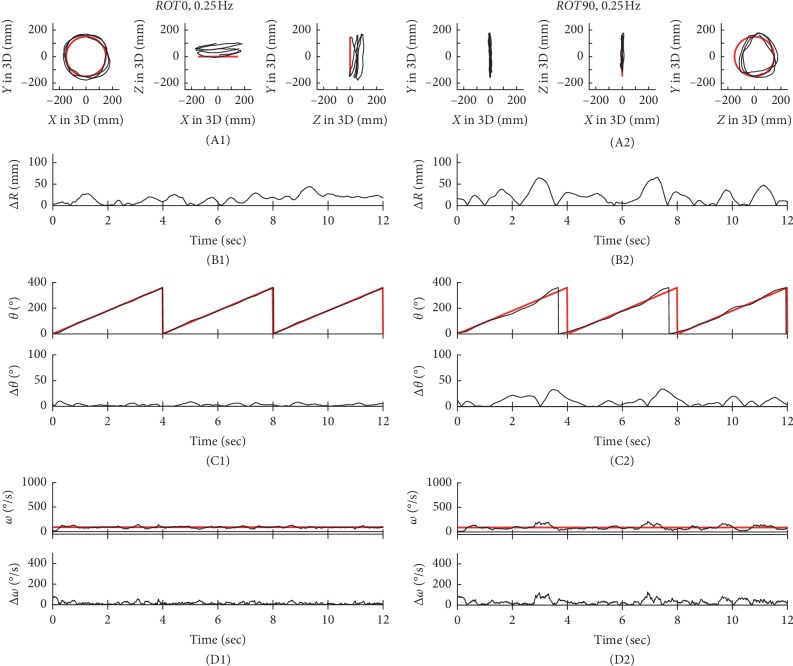
Typical examples of the tracking movement at 0.25 Hz. The trajectories of circular tracking movements for 0.25 Hz in the frontal plane (*ROT0*, A1) and the sagittal plane (*ROT90*, A2). Absolute values of Δ*R* for *ROT0* (B1) and *ROT90* (B2). *θ* and absolute values of Δ*θ* for *ROT0* (C1) and *ROT90* (C2). Absolute values of Δ*ω* or *ROT0* (D1) and *ROT90* (D2).

**Figure 4:**
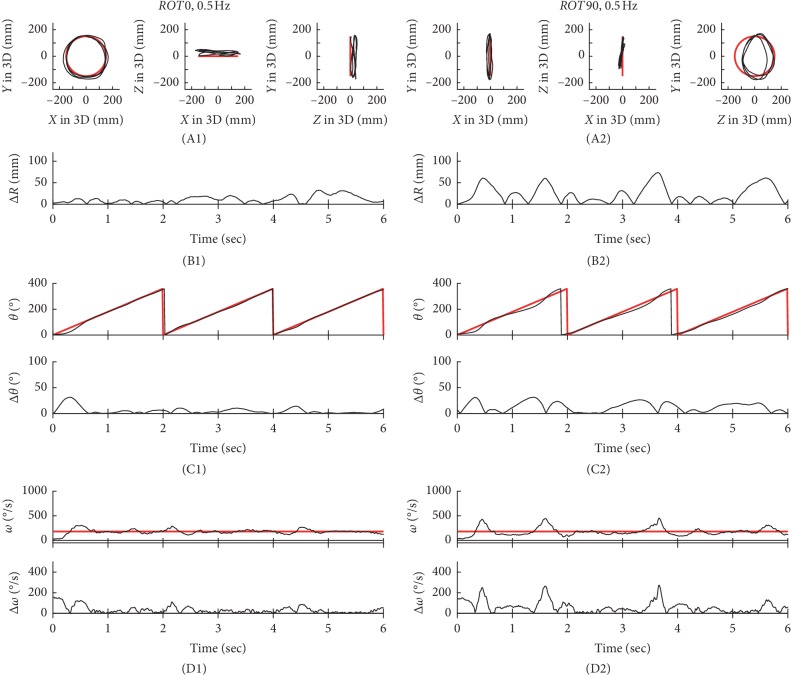
Typical examples of the tracking movement at 0.5 Hz. The trajectories of circular tracking movements for 0.5 Hz in the frontal plane (*ROT0*, A1) and the sagittal plane (*ROT90*, A2). Absolute values of Δ*R* for *ROT0* (B1) and *ROT90* (B2). *θ* and absolute values of Δ*θ* for *ROT0* (C1) and *ROT90* (C2). Absolute values of Δ*ω* for *ROT0* (D1) and *ROT90* (D2).

**Figure 5:**
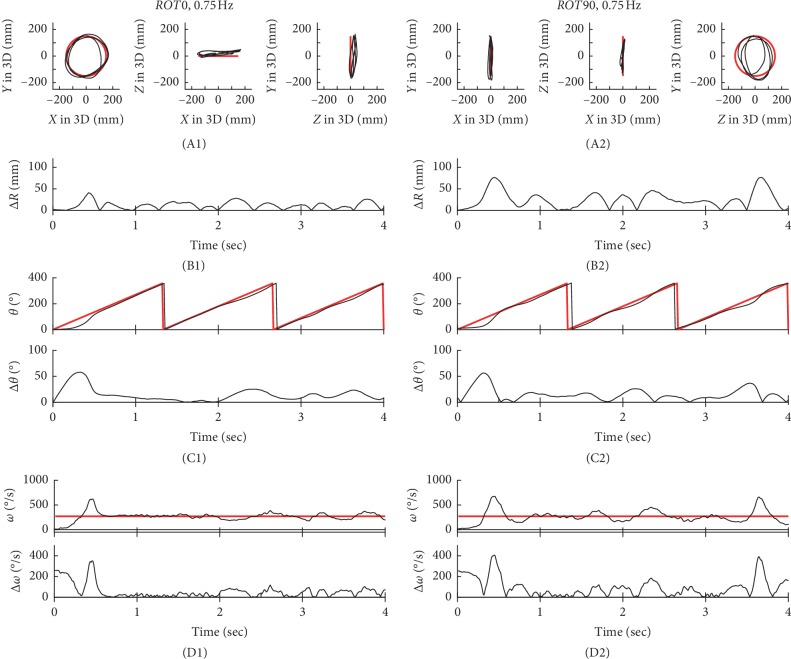
Typical examples of the tracking movement at 0.75 Hz. The trajectories of circular tracking movements for 0.75 Hz in the frontal plane (*ROT0*, A1) and the sagittal plane (*ROT90*, A2). Absolute values of Δ*R* for *ROT0* (B1) and *ROT90* (B2). *θ* and absolute values of Δ*θ* for *ROT0* (C1) and *ROT90* (C2). Absolute values of Δ*ω* for *ROT0* (D1) and *ROT90* (D2).

In Figures 2–5, typical examples of circular tracking movements can be seen during monocular vision at four target speeds (0.125, 0.25, 0.5, and 0.75 Hz). Figures 2(A1), 3(A1), 4(A1), and 5(A1) show the trajectories of the circular tracking movements in the frontal plane at each target speed, while Figures 2(A2), 3(A2), 4(A2), and 5(A2) show the trajectories (black lines) of the circular tracking movements in the sagittal plane at each target speed. During circular tracking movement in both frontal and sagittal planes, the trajectory variability did not trend towards an increase with increasing target speed. More variability in the sagittal plane was seen at each target speed ((B1) and (B2)) in Figures 2–5.

**Figure 6:**
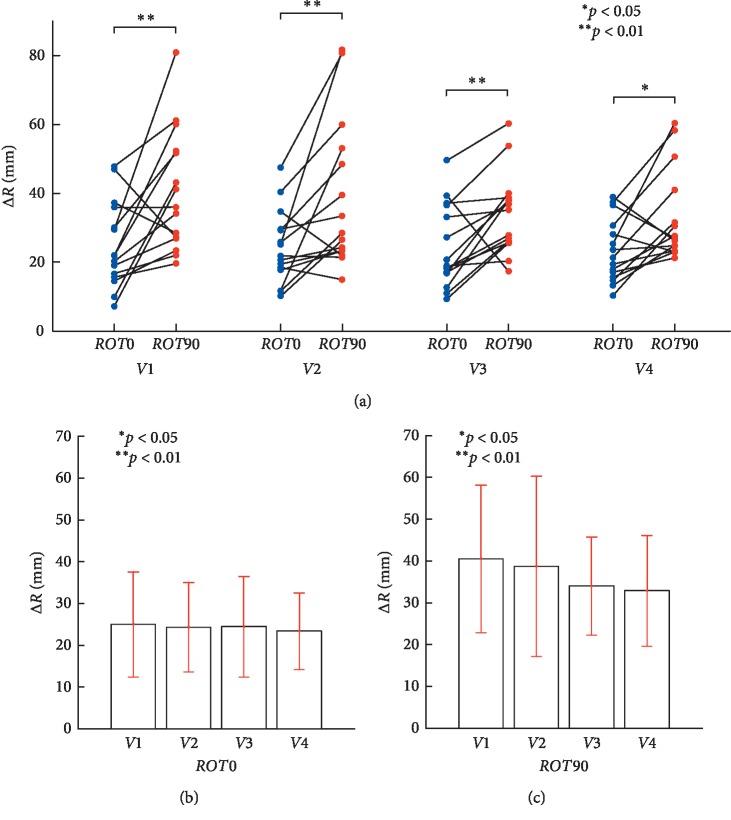
Evaluation of the circular tracking performance based on Δ*R*. (a) Pairwise comparisons of Δ*R* analyzing the speed effect between *ROT0* and *ROT90*. (b) Pairwise comparisons are indicated for Δ*R*, in the frontal plane *ROT0*, at four target speeds (Item C in Table 1). Δ*R* was 24.97 ± 12.56 mm for 0.125 Hz, 24.26 ± 10.69 mm for 0.25 Hz, 24.4 ± 12.03 mm for 0.5 Hz, and 23.35 ± 9.21 mm for 0.75 Hz, respectively. (c) Pairwise comparisons are displayed for Δ*R*, in the sagittal plane *ROT90*, at four target speeds (Item D in Table 1). Δ*R* was 40.54 ± 17.64 mm for 0.125 Hz, 38.74 ± 21.52 mm for 0.25 Hz, 34.01 ± 11.75 mm for 0.5 Hz, and 32.89 ± 13.23 mm for 0.75 Hz, respectively.

We first examined the circular movement in 3D space at the four target speeds using Δ*R*. There was a significant effect of plane (plane: *F* (1, 14) = 18.367, *p*=0.001, partial *η*^2^ = 0.567; Item A in Table 1). Frontal and sagittal planes differentially affected the performance of Δ*R* during monocular vision.  Figure 6(a) shows the pairwise comparison for a main effect of movement planes corrected using a Bonferroni adjustment. The differences in Δ*R* are statistically significant under the conditions of *V*1 (*r* = 0.644, *p* < 0.01), *V*2 (*r* = 0.637, *p* < 0.01), *V*3 (*r* = 0.658, *p* < 0.01), and *V*4 (*r* = 0.604, *p* < 0.05) between *ROT0* and *ROT90* (Item B in Table 1). This suggests the subjects found it more difficult to track the target radius in the sagittal plane (*M* = 36.55 mm, SE = 3.64 mm) than in the frontal plane (*M* = 24.25 mm, SE = 2.3 mm), when the target speed was over 0.125 Hz.

However, the effect of speed was not significant (speed: *F*(2.153, 30.148) = 1.781, *p*=0.184, partial *η*^2^ = 0.113; Item A in Table 1). Furthermore, there was no significant interaction between the factors of plane and speed (*F* (1.986, 27.806) = 0.905, *p*=0.415, partial *η*^2^ = 0.061; Item A in Table 1). As shown in Figures 6(b) and 6(c), we found that the variability of Δ*R* with respect to both planes remains constant as the target velocity increases during circular tracking movement and monocular vision. This suggests the differences in Δ*R* are mediated through different movement planes rather than different tracking speeds.

### 3.2. Differences in Performance Based on Δθ

Figures 2(C1), 3(C1), 4(C1), and 5(C1) show *θ* in polar coordinates (top trace) and Δ*θ* (bottom trace) in the frontal plane at the four target speeds (0.125, 0.25, 0.5, and 0.75 Hz). Similarly, Figures 2(C2), 3(C2), 4(C2), and 5(C2) show *θ* in polar coordinates (top trace) and Δ*θ* (bottom trace) in the sagittal plane at each target speed.

**Figure 7:**
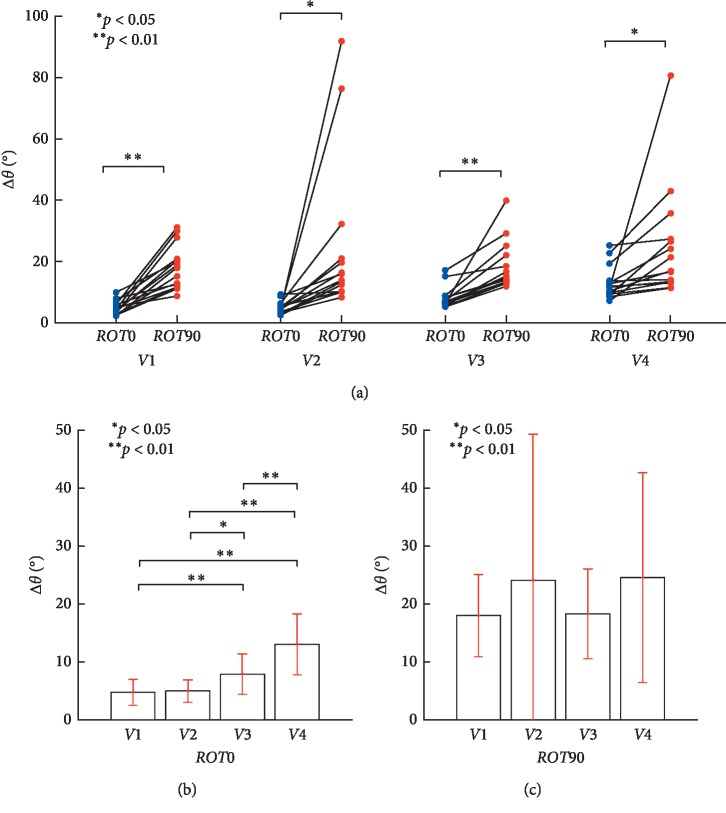
Evaluation of the circular tracking performance based on Δ*θ*. (a) Pairwise comparisons of Δ*θ* analyzing the speed effect between *ROT0* and *ROT90*. (b) Pairwise comparisons are indicated for Δ*θ*, in the frontal plane *ROT0*, at four target speeds (*n* = 15). Δ*θ* was 4.77 ± 2.24° for 0.125 Hz, 4.98 ± 1.94° for 0.25 Hz, 7.91 ± 3.5° for 0.5 Hz, and 13.02 ± 5.26° for 0.75 Hz, respectively. (c) Pairwise comparisons are displayed for Δ*θ*, in the sagittal plane *ROT90*, at four target speeds (*n* = 15). Δ*θ* was 17.98 ± 7.12° for 0.125 Hz, 24.09 ± 25.26° for 0.25 Hz, 18.29 ± 7.76° for 0.5 Hz, and 24.54 ± 18.11° for 0.75 Hz, respectively.

We compared Δ*θ* between the frontal and sagittal planes at each target speed to investigate the differences in position angle during monocular visually guided tracking movements. There was a significant effect of plane (plane: *F*(1, 14) = 15.653, *p*=0.001, partial *η*^2^ = 0.528; Item A in Table 2).  Figure 7(a) shows the pairwise comparison for the effect of different movement planes corrected using a Bonferroni adjustment. The differences of Δ*θ* are statistically significant under the conditions of *V*1(*r* = 0.882, *p* < 0.01), *V*2 (*r* = 0.613, *p* < 0.01), *V*3 (*r* = 0.825, *p* < 0.01), and *V*4 (*r* = 0.567, *p* < 0.05) between *ROT0* and *ROT90* (Item B in Table 2). There was a significant difference in Δ*θ* with respect to the accuracy of circular tracking in *ROT0* and *ROT90* when the speed was over 0.125 Hz. This result suggests the subjects found it more difficult to synchronize the target position and the tracer in the sagittal plane (*M* = 21.23°, SE = 3.46°) than in the frontal plane (*M* = 7.67°, SE = 0.714°) at all target speeds.

Furthermore, there was a significant effect of speed (speed: *F*(1.781, 24.94) = 4.51, *p*=0.025, partial *η*^2^ = 0.244; Item A in Table 2). We also examined the relationship between Δ*θ* and target speed in each plane. A pairwise comparison (Bonferroni correction) was performed for Δ*θ* in the frontal (*ROT0*) and sagittal (*ROT90*) planes, at four target speeds (*n* = 15). As shown in Figure 7(b), the differences in Δθ between different target speeds were significant under the conditions *V*1: *V*3 (*r* = 0.783, *p* < 0.01), *V*1: *V*4 (*r* = 0.881, *p* < 0.01), *V*2: *V*3 (*r* = 0.711, *p* < 0.05), *V*2: *V*4 (*r* = 0.852, *p* < 0.01), and *V*3: *V*4 (*r* = 0.888, *p* < 0.01) in the *ROT0* plane (Item C in Table 2). In the frontal plane *ROT0*, Δ*θ* increased with the target speed. This indicates that phase control of circular tracking movement in the frontal plane *ROT0* as the speed increases becomes more difficult. As shown in Figure 7(c), the differences in Δ*θ* between the target speeds are not significant in the *ROT90* plane (Item D in Table 2). Likewise, there was no difference of Δ*θ* in the sagittal plane *ROT90* as the target speed increased. This demonstrates the difficulty in synchronizing the target and tracer positions in the sagittal plane *ROT90* regardless of the target speed. The interaction between the plane and speed factors was not significant (*F* (1.685, 23.597) = 1.920, *p*=0.173, partial *η*^*2*^ = 0.121; Item A in Table 2).

### 3.3. Differences in Performance Based on Δ*ω*

In Figures 2(D1), 3(D1), 4(D1), and 5(D1), the effect of 4 different speeds (*V*1: 0.125 Hz (*n* = 15), *V*2: 0.25 Hz (*n* = 15), *V*3: 0.5 Hz (*n* = 15), and *V*4: 0.75 Hz (*n* = 15)) on Δ*ω* in the frontal plane can be seen. Similarly, Figures 2(D2), 3(D2), 4(D2), and 5(D2) show Δ*ω* (bottom trace) in the sagittal plane at each target speed.

The frontal and sagittal planes were compared and Δ*ω* evaluated at each target speed to investigate velocity-control accuracy. Both plane and speed were seen to have a significant effect (plane: *F*(1, 15) = 171.36, *p*=0, partial *η*^2^ = 0.92; speed: *F*(1.07, 16.03) = 252.33, *p*=0, partial *η*^2^ = 0.944; Item A in Table 3). There was also a significant interaction between plane and speed (*F* (1.258, 18.87) = 38.1, *p*=0, partial *η*^2^ = 0.717; Item A in Table 3). Here, significant effects of plane, speed, and an interaction between plane and speed were seen in Δ*ω* during circular tracking. An interaction between target speed and depth during 3D target-tracking movements would affect the ability of Δ*ω* to evaluate velocity-control precision during circular tracking movements.

**Figure 8:**
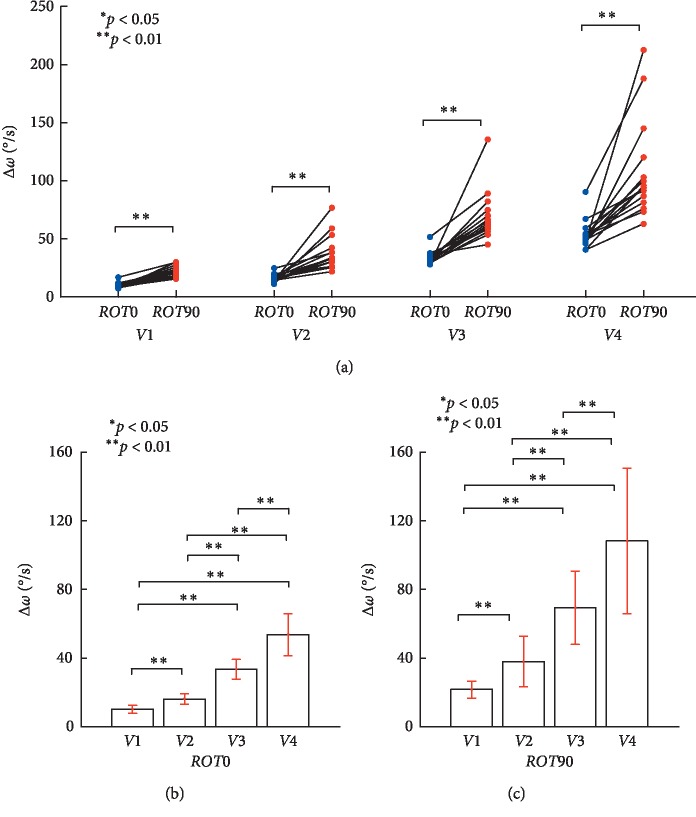
Evaluation of the circular tracking performance based on Δ*ω*. (a) Pairwise comparisons of Δ*ω* analyzing the speed effect between *ROT0* and *ROT90*. (b) Pairwise comparisons are shown for Δ*ω*, in the frontal plane *ROT0*, at four target speeds (*n* = 15). Δ*ω* was 10.14 ± 2.33° s^−1^ for 0.125 Hz, 16.12 ± 3.15° s^−1^ for 0.25 Hz, 33.54 ± 5.81° s^−1^ for 0.5 Hz, and 53.7 ± 12.12° s^−1^ for 0.75 Hz, respectively. (c) Pairwise comparisons were performed for Δ*ω*, in the sagittal plane *ROT90* at four target speeds (*n* = 15). Δ*ω* was 21.62 ± 4.85° s^−1^ for 0.125 Hz, 37.93 ± 14.83° s^−1^ for 0.25 Hz, 69.34 ± 21.43° s^−1^ for 0.5 Hz, and 108.31 ± 42.37° s^−1^ for 0.75 Hz, respectively.

As shown in Figure 8(a), the differences in Δ*ω* were statistically significant under the conditions *V*1 (*r* = 0.922, *p* < 0.01), *V*2 (*r* = 0.815, *p* < 0.01), *V*3 (*r* = 0.857, *p* < 0.01), and *V*4 (*r* = 0.82, *p* < 0.01) between *ROT0* and *ROT90* (Item B in Table 3). This result suggests the subjects were more accurate in the frontal plane (*M* = 28.38° s^−1^, SE = 1.43° s^−1^) when compared to the sagittal plane (*M* = 59.3° s^−1^, SE = 4.94° s^−1^) when synchronizing the angular velocities of the target and tracer.

Next, the relationship between Δ*ω* and the target speed for both planes was examined. As shown in Figure 8(b), a pairwise comparison (Bonferroni correction) was performed for Δ*ω*, in the frontal (*ROT0*) and sagittal planes (*ROT90*), at four target speeds (*n* = 15). The differences in Δ*ω* at the target speeds were found to be statistically significant under the following conditions: *V*1: *V*2 (*r* = 0.976, *p* < 0.01), *V*1: *V*3 (*r* = 0.986, *p* < 0.01), *V*1: *V*4 (*r* = 0.974, *p* < 0.01), *V*2: *V*3 (*r* = 0.978, *p* < 0.01), *V*2: *V*4 (*r* = 0.97, *p* < 0.01), and *V*3: *V*4 (*r* = 0.942, *p* < 0.01) for the *ROT0* plane (Item C in Table 3). Like Δ*θ*, Δ*ω* increased in the frontal plane (*ROT0*) as target speed increased. This indicates that it is difficult to control the velocity of circular tracking movement in the frontal plane (*ROT0*) as the speed increases. Also, there was a significant difference in Δ*ω* at the target speeds under the following conditions: *V*1: *V*2 (*r* = 0.828, *p* < 0.01), *V*1: *V*3 (*r* = 0.939, *p* < 0.01), *V*1: *V*4 (*r* = 0.916, *p* < 0.01), *V*2: *V*3 (*r* = 0.931, *p* < 0.01), *V*2: *V*4 (*r* = 0.899, *p* < 0.01), and *V*3: *V*4 (*r* = 0.835, *p* < 0.01) for the *ROT90* plane (Item D in Table 3).

## 4. Discussion

In this study, we quantitatively evaluated the motor control characteristics of circular tracking movements during monocular vision in a 3D VR space. We analyzed the spatiotemporal relationship during monocular vision between circular tracking movements and the target motion at various speeds in two different rotation axes.

We found that Δ*ω*, which describes temporal errors during motor control in polar coordinates, increased in both the frontal and sagittal planes when the target speed increased. This suggests that, irrespective of the target's rotation axis in 3D space, an increasing target speed makes it more difficult to synchronize angular velocities of the target and the tracer ((B) and (C) in Figure 8), whereas, Δ*R*, which indicates spatial errors of motor control in polar coordinates, did not increase in either the frontal or sagittal planes irrespective of target speed. This suggests that, irrespective of the target's rotation axis, the target speed has no effect on spatial tracking of the target ((B) and (C) in Figure 6). Furthermore, as the target speed increases, Δ*θ* increases in the frontal plane (Figure 7(b)), whereas Δ*θ* becomes constant at approximately 21° in the sagittal plane. Regardless of the target speed, phase control accuracy in the frontal plane was seen to increase 2.8-fold in the sagittal plane.

The results show that, during 3D circular tracking movement and monocular vision, motor control in position and velocity in the frontal plane is twice as accurate as that of the sagittal plane. It was also shown that Δ*R* remains constant with respect to rotation plane rather than the target speed. Furthermore, in the sagittal plane, Δ*θ* became constant at approximately 21° regardless of the target speed. The difference in Δ*θ* was found to be dependent on plane orientation during circular tracking movements.

### 4.1. Effect of Depth and Target Speed

The visuomotor system primarily uses visual input for reference, followed by central processing of this input and subsequent muscular innervation to generate movement. It is known that humans can recognize depth information through monocular vision, as well as binocular vision.

Many studies have compared monocular and binocular vision during reaching and grasping movements. Visuomotor movement during monocular vision is associated with lower accuracy [24], difficulties in initial visuomotor movement setup [22], underestimation of distance [21], and slower task execution [20, 26] when compared to binocular vision. It has been reported that motor control performance decreases with increasing visuomotor movement during monocular vision when compared to binocular vision [36]. This can, at least in part, be explained by insufficient depth information such as binocular disparities during monocular vision.

In this study, we verified that depth information during monocular vision can be acquired by occlusion of target and tracer. The average Δ*R* was 24.25 mm and 36.55 mm in the frontal (*ROT0*) and sagittal planes (*ROT90*) from Figures 6(b) and 6(c), respectively. There were significant differences in Δ*R* in *ROT0* and *ROT90* at various velocities (Item B in Table 1). We show that tracking movement is possible while maintaining a fixed distance from the center of a circular movement depending on depth information but regardless of the velocity. Our data show that Δ*R* in *ROT0* is smaller than that of *ROT90*, which indicates inaccurate visuomotor movement at *ROT90*. This result is consistent with previous reports describing visual feedback for limb position is most accurate in the azimuth and least accurate in the direction of depth [37–42].

We also found Δ*θ* increases with target velocity during circular tracking movement within the frontal plane (Figure 7(b)). Conversely, in the sagittal plane, Δ*θ* becomes constant at approximately 21°. Irrespective of the target speed, phase control in the frontal plane is 2.8 times more accurate than that in the sagittal plane.

As shown in Figure 7(a), Δ*θ* increases with respect to velocity at *V*1 to *V*4 in *ROT0*, this can be interpreted as the subject performing feedback control based on visual information. We have also considered the subject may have performed feedback and feedforward control in *ROT90*, which resulted in a constant Δ*θ* from 21° regardless of the velocity change. Even during monocular vision, the visual feedback of limb position is most accurate in the azimuth and least accurate in the direction of depth. At the velocity of *V*3 and *V*4, the subject cannot clearly recognize the location of the target, and we suggest the circular tracking movement is performed through feedforward control. Feedforward control dominated at high target frequencies [6]. The larger Δ*θ* at *V*2 than at *V*3 may indicate target localization is performed using feedback control [12].

During control of angular velocity, as the target velocity increases, Δ*ω* in *ROT0* and *ROT90* increases accordingly (Figures 8(b) and 8(c)). The discrepancy between monocular and binocular vision in reaching and grasping movement in a real environment (i.e., not VR as examined here) has reportedly been dominated by binocular vision (2.5- to 3-fold) for each movement [24]. During monocular vision, speed control at *ROT0* was approximately 2.1 times more accurate than that at *ROT90*. The increase of *ω* indicates that, during faster velocities, the subjects struggled to track the object accurately. During monocular vision, a delay in circular tracking movements can occur due to a gaze shift, as opposed to binocular vision where this effect is not as great, and therefore, Δ*ω* increases in line with the speed [25, 43]. Also, we can infer that Δ*ω* is larger in the *ROT90* during monocular vision when the depth of the target cannot be accurately gauged [21].

### 4.2. Characteristics of Tracking Movement during Monocular Vision and Its Application

There are a limited number of studies which quantitatively investigate monocular visually guided circular tracking movement in a 3D VR environment. Here, we present a study examining this in both *ROT0* and *ROT90* using the previously outlined parameters in polar coordinates.

By analyzing the parameters of Δ*R*, Δ*θ*, and Δ*ω*, we have shown that, during monocular vision, there is a smaller error rate in each parameter at *ROT0* than at *ROT90*. As monocular vision at *ROT90* provides a less reliable input regarding object location and features, it is possible that the ability to use predictive control during action sequences may be reduced. This may lead to a delay in the initiation of a subsequent action phase [25, 43, 44].

With respect to the parameter of Δ*θ*, the control position was aligned using visual feedback at *ROT0*. However, the control position was aligned using feedforward control when the speed was over 0.5 Hz at *ROT90*.

The field of robotics, in particular, is actively researching 3D depth estimation based on monocular vision [45, 46]. To estimate depth from images, several monocular (single image) cues such as texture variations, texture gradients, interposition, occlusion, known object sizes, light and shading, hazing, and defocus are used. Distance, position, and velocity of motor control at *ROT0* and *ROT90* in monocular vision are the basic information for the construction of a monocular visuomotor system in the field of robotics.

The balance of binocular vision will collapse, if it becomes amblyopia or strabismus in one eye. It results in improper binocular vision including binocular instability and fixation disparity. In particular, the improper binocular vision starts with dyslexia in our lives and adversely affects the visuomotor control in 3D space [47–52]. However, according to recent researches, motor control and/or learning in monocular vision may be the key to correct the unbalance of binocular vision by the amblyopia and strabismus. In other words, it is suggested that motor control and/or learning in monocular vision (monocular occlusion) may help dyslexic children to develop reliable vergence control, thereby improving their reading skills [53, 54]. In the future, the method and analysis proposed in this study will be applicable to examine the effectiveness of the monocular occlusion in terms of motor control, in particular, the position and velocity controls. Also, monopsia is a condition where people cannot perceive in 3D even though their eyes are clinically healthy. The results of this study could be used as preliminary data in the analysis of visuomotor control in the monopsia.

Moreover, Iriki et al. studied behavioral effects of tool use in humans and monkeys [55–57]. They reported that body representation in the brain could be changed following tool use. Body image has been extended to the tool. For further study, we will quantitatively analyze the change of arm kinematics in VR space under the condition of displaying the VR stick and 3D hand and not displaying hand information.

## 5. Conclusion

In this study, we have analyzed the motor control characteristic of circular tracking movements during monocular vision in a 3D VR space. It is found that temporal errors were proportional to the change of target speed, whereas spatial errors were influenced by the depth cues instead of the target speed. We considered that the subject performed feedback control based on visual information in the frontal plane *ROT0*. On the other hand, the subject performed feedback and feedforward control in the sagittal plane *ROT90*. Moreover, both temporal and spatial errors of the circular tracking movement in the frontal plane, which requires less depth information, were lower than that in the sagittal plane. The increase in errors during circular tracking movements with respect to depth indicated that the lack of depth information during monocular vision causes circular tracking movement in the sagittal plane less accurate.

## Figures and Tables

**Table 1:** A summary of the statistical analysis of Δ*R*.

Item	Variable	Test	Statistic	Confidence
A	Δ*R* between the frontal and sagittal planes at each target speed	Two-way repeated-measures ANOVA	Plane: Mauchly's test *χ*^2^(0) = 0, *p* = nothing, *ε* = 1; *F*(1, 14) = 18.367 Speed: Mauchly's test *χ*^2^(5) = 16.23, *p* = 0.006, *ε* = 0.718; *F*(2.153, 30.148) = 1.781 Interaction: Mauchly's test *χ*^2^(5) = 11.354, *p* = 0.045, *ε* = 0.662; *F*(1.986, 27.806) = 0.905	Plane: *p* = 0.001, partial *η*^2^ = 0.567, power = 1.0, corrected by Greenhouse–Geisser Speed: *p* = 0.184, partial *η*^2^ = 0.113, power = 1.0, corrected by Greenhouse–Geisser Interaction: *p* = 0.415, partial *η*^2^ = 0.061, power = 0.64, corrected by sphericity assumed

B	Δ*R* under the conditions of *V*1, *V*2, *V*3, and *V*4 between *ROT0* and *ROT90*	Bonferroni-corrected pairwise comparisons	*V*1 between *ROT0* and *ROT90*: *t*(14) = 3.149 *V*2 between *ROT0* and *ROT90*: *t*(14) = 3.088 *V*3 between *ROT0* and *ROT90*: *t*(14) = 3.273 *V*4 between *ROT0* and *ROT90*: *t*(14) = 2.837	*V*1 between *ROT0* and *ROT90*: *p* = 0.007, CI = −26.172∼−4.966, *r* = 0.644 *V*2 between *ROT0* and *ROT90*: *p* = 0.008, CI = −24.525∼−4.421, *r* = 0.637 *V*3 between *ROT0* and *ROT90*: *p* = 0.006, CI = −15.908∼−3.313, *r* = 0.658 *V*4 between *ROT0* and *ROT90*: *p* = 0.013, CI = −16.76∼−2.329, *r* = 0.604

C	Δ*R* of target speeds under the conditions of *V*1 : *V*2, *V*1 : *V*3, *V*1 : *V*4, *V*2 : *V*3, *V*2 : *V*4, and *V*3 : *V*4 on the *ROT0* phase	Bonferroni-corrected pairwise comparisons	*V*1 : *V*2: *t* (14) = 0.202 *V*1 : *V*3: *t* (14) = 0.163 *V*1 : *V*4: *t* (14) = 0.528 *V*2 : *V*3: *t* (14) = 0.057 *V*2 : *V*4: *t* (14) = 0.351 *V*3 : *V*4: *t* (14) = 0.644	*V*1 : *V*2: *p* = 1, CI = −10.049–11.464, *r* = 0.054 *V*1 : *V*3: *p* = 1, CI = −10.083–11.218, *r* = 0.044 *V*1 : *V*4: *p* = 1, CI = −7.808–11.055, *r* = 0.14 *V*2 : *V*3: *p* = 1, CI = −7.671–7.391, *r* = 0.015 *V*2: *V*4: *p* = 1, CI = −7.098–8.929, *r* = 0.093 *V*3 : *V*4: *p* = 1, CI = −3.98–6.092, *r* = 0.17

D	Δ*R* of target speeds under the conditions of *V*1 : *V*2, *V*1 : *V*3, *V*1 : *V*4, *V*2 : *V*3, *V*2 : *V*4, and *V*3 : *V*4 on the *ROT90* phase	Bonferroni-corrected pairwise comparisons	*V*1 : *V*2: *t* (14) = 0.619 *V*1 : *V*3: *t* (14) = 2.26 *V*1 : *V*4: *t* (14) = 1.65 *V*2 : *V*3: *t* (14) = 1.438 *V*2 : *V*4: *t* (14) = 1.206 *V*3 : *V*4: *t* (14) = 0.503	*V*1 : *V*2: *p* = 1, CI = −7.131–10.738, *r* = 0.163 *V*1 : *V*3: *p* = 0.242, CI = −2.336–15.387, *r* = 0.517 *V*1 : *V*4: *p* = 0.727, CI = −6.578–21.874, *r* = 0.403 *V*2 : *V*3: *p* = 1, CI = −5.355–14.799, *r* = 0.359 *V*2 : *V*4: *p* = 1, CI = −9.031–20.72, *r* = 0.307 *V*3 : *V*4: *p* = 1, CI = −5.72–7.965, *r* = 0.133

**Table 2:** A summary of the statistical analysis of Δ*θ*.

Item	Variable	Test	Statistic	Confidence
A	Δ*θ* between the frontal and sagittal planes at each target speed	Two-way repeated-measures ANOVA	Plane: Mauchly's test *χ*^2^(0) = 0, *p* = nothing, *ε* = 1; *F*(1, 14) = 15.653 Speed: Mauchly's test *χ*^2^(5) = 30.566, *p* = 0, *ε* = 0.594; *F*(1.781, 24.940) = 4.511 Interaction: Mauchly's test *χ*^2^(5) = 32.299, *p* = 0*ε* = 0.562; *F*(1.685, 23.597) = 1.920	Plane: *p* = 0.001, partial *η*^2^ = 0.528, power = 1.0, corrected by Greenhouse–Geisser Speed: *p* = 0.025, partial *η*^2^ = 0.244, power = 1.0, corrected by Greenhouse–Geisser Interaction: *p* = 0.173, partial *η*^2^ = 0.121, power = 0.94, corrected by Greenhouse–Geisser

B	Δ*θ* under the conditions of *V*1, *V*2, *V*3, and *V*4 between *ROT0* and *ROT90*	Bonferroni-corrected pairwise comparisons	*V*1 between *ROT0* and *ROT90*: *t* (14) = 7.007 *V*2 between *ROT0* and *ROT90*: *t* (14) = 2.903 *V*3 between *ROT0* and *ROT90*: *t* (14) = 5.452 *V*4 between *ROT0* and *ROT90*: *t* (14) = 2.578	*V*1 between *ROT0* and *ROT90*: *p* = 0, CI = −17.262∼−9.171, *r* = 0.882 *V*2 between *ROT0* and *ROT90*: *p* = 0, CI = −33.213∼−4.991, *r* = 0.613 *V*3 between *ROT0* and *ROT90*: *p* = 0, CI = −14.475∼−6.302, *r* = 0.825 *V*4 between *ROT0* and *ROT90*: *p* = 0.022, CI = −21.097∼−1.936, *r* = 0.567

C	Δ*θ* of target speeds under the conditions of *V*1 : *V*2, *V*1 : *V*3, *V*1 : *V*4, *V*2 : *V*3, *V*2 : *V*4, and *V*3 : *V*4 on the *ROT0* phase	Bonferroni-corrected pairwise comparisons	*V*1 : *V*2: *t* (14) = 0.562 *V*1 : *V*3: *t* (14) = 4.704 *V*1 : *V*4: *t* (14) = 6.976 *V*2 : *V*3: *t* (14) = 3.782 *V*2 : *V*4: *t* (14) = 6.1 *V*3 : *V*4: *t* (14) = 7.214	*V*1 : *V*2: *p* = 1, CI = −1.404–0.97, *r* = 0.148 *V*1 : *V*3: *p* = 0.002, CI = −5.188∼−1.091, *r* = 0.783 *V*1 : *V*4: *p* = 0, CI = −11.89∼−4.625, *r* = 0.881 *V*2 : *V*3: *p* = 0.012, CI = −5.293∼−0.551, *r* = 0.711 *V*2 : *V*4: *p* = 0, CI = −12.085∼−3.996, *r* = 0.852; *V*3 : *V*4: *p* = 0, CI = −7.295∼−2.941, *r* = 0.888

D	Δ*θ* of target speeds under the conditions of *V*1 : *V*2, *V*1 : *V*3, *V*1 : *V*4, *V*2 : *V*3, *V*2 : *V*4, and *V*3 : *V*4 on the *ROT90* phase	Bonferroni-corrected pairwise comparisons	*V*1 : *V*2: *t* (14) = 1.154 *V*1 : *V*3: *t* (14) = 0.199 *V*1 : *V*4: *t* (14) = 1.647 *V*2 : *V*3: *t* (14) = 1.154 *V*2 : *V*4: *t* (14) = 0.114 *V*3 : *V*4: *t* (14) = 2.199	*V*1 : *V*2: *p* = 1, CI = −22.337–10.131, *r* = 0.295 *V*1: *V*3: *p* = 1, CI = −5.121–4.497, *r* = 0.053 *V*1: *V*4: *p* = 0.73, CI = −18.774–5.658, *r* = 0.403 *V*2: *V*3: *p* = 1, CI = −9.61–21.193, *r* = 0.295 *V*2: *V*4: *p* = 1, CI = −12.673–11.764, *r* = 0.03 *V*3: *V*4: *p* = 0.271, CI = −14.964–2.472, *r* = 0.507;

**Table 3:** A summary of the statistical analysis of Δ*ω*.

Item	Variable	Test	Statistic	Confidence
A	Δ*ω* between the frontal and sagittal planes at each target speed	Two-way repeated-measures ANOVA	Plane: Mauchly's test *χ*^2^(0) = 0, *p* = nothing, *ε* = 1; *F*(1, 14) = 39.925 Speed: Mauchly's test *χ*^2^(5) = 42.467, *p* = 0, *ε* = 0.392; *F*(1.177, 16.485) = 114.229 Interaction: Mauchly's test *χ*^2^(5) = 29.58, *p* = 0, *ε* = 0.446; *F*(1.339, 18.743) = 17.509	Plane: *p* = 0, partial *η*^2^ = 0.74, power = 1.0, corrected by Greenhouse–Geisser Speed: *p* = 0, partial *η*^2^ = 0.891, power = 1.0, Corrected by Greenhouse–Geisser Interaction: *p* = 0, partial *η*^2^ = 0.556, power = 1.0, corrected by Greenhouse–Geisser

B	Δ*ω* under the conditions of *V*1, *V*2, *V*3, and *V*4 between *ROT0* and *ROT90*	Bonferroni-corrected pairwise comparisons	*V*1 between *ROT0* and *ROT90*: *t* (14) = 8.937 *V*2 between *ROT0* and *ROT90*: *t* (14) = 5.272 *V*3 between *ROT0* and *ROT90*: *t* (14) = 6.224 *V*4 between *ROT0* and *ROT90*: *t* (14) = 5.363	*V*1 between *ROT0* and *ROT90*: *p* = 0, CI = −14.233∼−8.724, *r* = 0.922 *V*2 between *ROT0* and *ROT90*: *p* = 0, CI = −30.686∼−12.938, *r* = 0.815 *V*3 between *ROT0* and *ROT90*: *p* = 0, CI = −48.137∼−23.465, *r* = 0.857 *V*4 between *ROT0* and *ROT90*: *p* = 0, CI = −76.445∼−32.766, *r* = 0.82

C	Δ*ω* of target speeds under the conditions of *V*1 : *V*2, *V*1 : *V*3, *V*1 : *V*4, *V*2 : *V*3, *V*2 : *V*4, and *V*3 : *V*4 on the *ROT0* phase	Bonferroni-corrected pairwise comparisons	*V*1 : *V*2: *t* (14) = 16.743 *V*1 : *V*3: *t* (14) = 22.196 *V*1 : *V*4: *t* (14) = 16.115 *V*2 : *V*3: *t* (14) = 17.705 *V*2 : *V*4: *t* (14) = 14.823 *V*3 : *V*4: *t* (14) = 10.479	*V*1 : *V*2: *p* = 0, CI = −7.081∼−4.888, *r* = 0.976; *V*1: *V*3: *p* = 0, CI = −26.636∼−20.165, *r* = 0.986 *V*1 : *V*4: *p* = 0, CI = −51.86∼−35.268, *r* = 0.974 *V*2 : *V*3: *p* = 0, CI = −20.435∼−14.397, *r* = 0.978 *V*2 : *V*4: *p* = 0, CI = −45.36∼−29.799, *r* = 0.97 *V*3 : *V*4: *p* = 0, CI = −26.068∼−14.258, *r* = 0.942

D	Δ*ω* of target speeds under the conditions of *V*1 : *V*2, *V*1 : *V*3, *V*1 : *V*4, *V*2 : *V*3, *V*2 : *V*4, and *V*3 : *V*4 on the *ROT90* phase	Bonferroni-corrected pairwise comparisons	*V*1 : *V*2: *t* (14) = 5.523 *V*1 : *V*3: *t* (14) = 10.194 *V*1 : *V*4: *t* (14) = 8.547 *V*2 : *V*3: *t* (14) = 9.539 *V*2 : *V*4: *t* (14) = 7.695 *V*3 : *V*4: *t* (14) = 5.675	*V*1 : *V*2: *p* = 0, CI = −25.384∼−7.251, *r* = 0.828 *V*1 : *V*3: *p* = 0, CI = −62.089∼−33.357, *r* = 0.939 *V*1 : *V*4: *p* = 0, CI = −117.816∼−55.566, *r* = 0.916 *V*2 : *V*3: *p* = 0, CI = −41.51∼−21.302, *r* = 0.931 *V*2 : *V*4: *p* = 0, CI = −98.439∼−42.307, *r* = 0.899 *V*3 : *V*4: *p* = 0, CI = −60.038∼−17.897, *r* = 0.835

## Data Availability

The data used to support the findings of this study are available from the corresponding author upon request.
